# The Roles of Salience and Value in Inattention Among Children With Attention-Deficit/Hyperactivity Disorder: A Pilot Investigation

**DOI:** 10.3389/fpsyg.2021.750525

**Published:** 2021-11-02

**Authors:** Erica D. Musser, Stephanie S. J. Morris, Kathleen Feeney, Rosario Pintos Lobo, Edward F. Ester

**Affiliations:** ^1^ABC ERICA Laboratory, Center for Children and Families, Department of Psychology, Florida International University, Miami, FL, United States; ^2^Department of Psychology and Institute of Neuroscience, University of Nevada, Reno, NV, United States

**Keywords:** attention-deficit/hyperactivity disorder, attention, reward, salience, value

## Abstract

Although inattention is a key symptom subdomain of attention-deficit/hyperactivity disorder (ADHD), the mechanisms underlying this subdomain and related symptoms remain unclear. There is a need for more granular approaches that allow for greater specificity in linking disruptions in specific domains of cognitive performance (e.g., executive function and reward processing) with behavioral manifestations of ADHD. Such approaches may inform the development of more targeted therapeutic interventions. Here, we describe the results of a pilot study of elementary-aged children (ages 6–12years) with ADHD (*n*=50) and typically developing children (*n*=48) utilizing a cognitive science task designed to target two dissociable mechanisms of attentional selection: a goal-driven mechanism (i.e., reward/value-driven) and a salience-driven mechanism. Participants were asked to optimally extract and combine information about stimulus salience and value to maximize rewards. While results of this pilot study are ambiguous due to the small sample size and limited number of task trials, data suggest that neither participants with ADHD nor typically developing participants performed optimally to maximize rewards, though typically developing participants were somewhat more successful at the task (i.e., more likely to report high-value targets) regardless of task condition. Further, the manuscript examines several follow-up questions regarding group differences in task response times and group differences in task performance as related to sustained attention across the duration of the task. Finally, the manuscript examines follow-up questions related to heterogeneity in the ADHD group (i.e., age, DSM 5 presentation, and comorbid diagnosis) in predicting task performance.

## Introduction

Attention-deficit/hyperactivity disorder (ADHD) is a complex neurodevelopmental disorder with a worldwide prevalence of 11% (Polanczyk et al., 2007; Visser et al., 2014). Although inattention is present among the majority of individuals with ADHD, the etiological sources of such behavioral symptoms remain unclear. The National Institute of Mental Health’s Research Domain Criteria (RDoC) initiative has called for a research-based framework for investigating mental disorders (Insel et al., 2010). RDoC redirects the primary focus of mental health research from behavioral features of disorders to the functioning of specific domains presumed to underlie such behavioral manifestations and symptoms (Cuthbert and Insel, 2013). These domains include as: negative and positive valence, cognitive, social, and arousal/regulatory systems (Insel, 2014).

Contemporary models of ADHD propose that behavioral symptoms of inattention and hyperactivity/impulsivity result from disruptions in core executive functions (i.e., cognitive domain) and/or reward processing (i.e., positive valence domain; Nigg, 2005; Willcutt et al., 2005). However, extant measures used to identify executive function deficits in ADHD have poor diagnostic reliability and meta-analytic work supports moderate effect sizes for associations between executive function subdomains and ADHD (*d*=0.46–0.69; Willcutt et al., 2005) with substantial heterogeneity across individuals (Fair et al., 2012). One explanation for these shortcomings is that common measures of executive function (e.g., Connors’ Continuous Performance Test) require complex coordination between multiple subcomponents of this construct (e.g., selective attention, working memory, and vigilance), making it difficult to conclusively link disruptions in one component with the behavioral symptoms of ADHD. Separately, a large body of empirical work has examined the role of reward processing among children with ADHD across a variety of reward-based tasks, with meta-analytic work revealing small-to-medium effect sizes (*d*=0.36–0.45) and substantial heterogeneity among individuals with ADHD (Marx et al., 2021).

To date, much of the work on ADHD has compared youth with and without ADHD on a single RDoC domain (e.g., examining selective attention independently of reward processing or vice versa). This makes it difficult to discern whether specific manifestations of ADHD behavioral symptomology result from disruptions in reward processing, selective attention, or a complex interaction of reward processing and selective attention. These limitations of the prior literature result in reduced understanding of ADHD etiology, as well as limitations in the identification of potential treatment targets. Thus, there is an urgent need for more granular approaches that will allow researchers to link disruptions in specific RDoC domains with different ADHD behavioral symptoms and presentations. A greater understanding of these links would enable enhanced diagnostic accuracy and could inform the development of more targeted therapeutic interventions.

Motivated by this need, the current study was designed to probe links between the domains of executive function and reward processing in a sample of children diagnosed with ADHD, as well as typically developing children. Following developments in the cognitive neuroscience literature (e.g., Navalpakkam et al., 2010), we take the view that subcomponents of executive function, such as selective attention, are intimately linked with reward processing and that disruptions in neurobiological systems supporting these domains contribute to ADHD behavioral symptomology.

### Optimal Deployment of Attention in ADHD

Humans need to make rapid choices among multiple targets embedded within noisy perceptual environments. Consider a student in a classroom deciding which visual stimuli to attend to. Valuable stimuli (e.g., a lesson at the chalk board) may be scarce or camouflaged by the other visual stimuli in the room, while less-valuable stimuli (e.g., colorful motivational posters on the walls) may be more plentiful and easier to find. To optimize the probability of taking in the lesson, the student must consider information about the salience and value of different targets. This raises a fundamental question: are rapid decisions among stimuli in cluttered environments dominated by sensory information (i.e., pursue the most salient stimuli), value information (i.e., pursue the more valuable stimuli), or a mixture of both?

Navalpakkam et al. (2010) addressed this question by requiring adult participants to search for two targets in a cluttered visual display while the relative value and salience of the targets were manipulated. Task performance was evaluated against three model benchmarks. The first model predicted that participants’ choices would be dominated by the perceptual properties (i.e., always attempt to select the most salient target; e.g., Irwin et al., 2000; Thompson and Bichot, 2005), the second model predicted that participants’ choices would be dominated by the economic properties (i.e., always attempt to select the most valuable target; Liston and Stone, 2008; Anderson et al., 2011; Anderson, 2016), and the third model predicted that participants’ choices would be guided by a Bayesian optimal combination of perceptual and economic information that would maximize reward over numerous trials (e.g., Najemnik and Geisler, 2005; Navalpakkam et al., 2009; Schutz et al., 2012). The Bayesian model systematically outperformed the perceptual and economic models, indicating that neurotypical adults incorporate and optimally combine information about the salience and value of stimuli when selecting information in cluttered environments.

Bayesian optimality provides a useful benchmark for examining the deployment of selective attention among children with ADHD. For example, a study design including manipulations of both salience and value would allow for the examination of deviations from optimal search behavior (e.g., purely salience-driven or purely value-driven) among children with ADHD, as well as for a more fine-grained characterization of the disorder rooted in cognitive science. This study represents a first step toward this goal. We hypothesized that typically developing children will exhibit optimal search behavior that maximizes rewards over trials while considering both salience and value of stimuli. In contrast, participants with ADHD will deviate from optimal search behavior either *via* a selective deficit in the processing of salience (i.e., a propensity to try to report the most valuable target in the display, even when it is less salient) or *via* a selective deficit in the processing of value (i.e., a propensity to report the most salient target in the display, even when it is of lesser value).

## Materials and Methods

### Participants

Ninety-eight child participants, ages six to twelve years (*M*=9.09, *SD*=1.77), completed the present study. Fifty met Diagnostic and Statistical Manual of Mental Disorders, fifth edition (DSM 5) criteria for ADHD (American Psychiatric Association, 2013), while 48 were typically developing comparison participants. Child participants were ethnically (i.e., 84.50% Hispanic/Latino) and racially (i.e., 11.50% African American, 1.00% Asian American, 85.40% White, and 2.10% identifying as “other race”) diverse, consistent with the demographics of the greater <<Masked for Review>>metro area. The 6 to 12year age range was chosen, as this is the most common period during which children are diagnosed with ADHD (Polanczyk et al., 2007) and allows for the specifications of DSM 5 that symptoms must be present prior to the age of 12years. This study was reviewed and approved by the Institutional Review Board (IRB) of Florida International University. Written informed consent and assent to participate in this study were provided by the participants’ legal guardian and by the participant, respectively.Full demographic information is presented in Table 1.

**Table 1:** Descriptive and diagnostic statistics for attention-deficit/hyperactivity disorder (ADHD) and typically developing (TD) groups.

Variable	TD (*n* =48)	ADHD (*n* =50)	*F/χ* ^2^	$\eta_{p}^{2}$/Cramer’s *V*
Demographics				
Age (years), mean (*SD*)	9.26 (1.89)	8.91 (1.63)	0.95	0.01
Gender (% male)	89.60	84.00	0.66	0.08
Ethnicity (% Hispanic/Latinx)	72.90	95.90	9.81^*^	0.32
Race				
African American (%)	14.60	8.30	0.92	0.01
Asian (%)	2.10	0.00	1.01	0.10
White (%)	81.30	89.60	1.34	0.12
Other race (%)	2.00	2.10	0.01	0.01
WASI-II FSIQ, mean (*SD*)	104.54 (13.08)	101.21 (17.10)	0.98	0.01
Stimulant medication (% receiving)	0.00	64.0	44.63^*^	0.68
Parent DBD-RS symptoms (*SD*)				
Inattention	0.08 (0.35)	6.40 (2.24)	372.43^*^	0.81
Hyperactivity/Impulsivity	0.13 (0.49)	4.86 (2.74)	138.72^*^	0.61
Total ADHD symptoms	0.21 (0.58)	11.15 (4.10)	335.00^*^	0.79
CD	0.00 (0.00)	0.36 (0.84)	9.05^*^	0.09
ODD	0.13 (0.49)	2.47 (2.07)	57.82^*^	0.39
Teacher DBD-RS symptoms (*SD*)				
Inattention	–	5.89 (2.60)	–	–
Hyperactivity/Impulsivity	–	4.24 (3.02)	–	–
Total ADHD symptoms	–	10.13 (4.45)	–	–
CD	–	0.49 (1.01)	–	–
ODD	–	2.42 (2.44)	–	–
Child Behavior Checklist				
Anxiety, T-score (*SD*)	51.17 (3.30)	58.96 (8.71)	29.72^*^	0.30

* *indicates p<0.05;*

*%, percentage; SD, Standard Deviation; WASI-II, Wechsler Abbreviated Scale of Intelligence-Second Edition; FSIQ, Full-Scale Intelligence Quotient; DBD-RS, Disruptive Behavior Disorder Rating Scale; ODD, Oppositional Defiant Disorder; CD, Conduct Disorder; Ethnicity and Race are treated as orthogonal constructs; and Missing data handled via list-wise deletion*.

### Recruitment and Identification

Participants were recruited through the university’s clinical treatment center and public advertising media (e.g., billboard, newspaper, and radio). Exclusion criteria included as: estimated Full-Scale Intellectual Quotient (IQ)<80; diagnosis of autism spectrum, cardiovascular, developmental, neurological, psychotic, or mood disorders; and use of psychotropic medication for disorders other than ADHD within the previous 6months. Additionally, participants were excluded if they had four to five symptoms of ADHD, as to be designated as typically developing, participants were required to have three or fewer inattentive or hyperactivity symptoms. In contrast, participants with ADHD were required to have six or more symptoms within either the inattentive or hyperactivity/impulsivity domains.

To determine eligibility, parents and teachers of participants with ADHD completed the Disruptive Behavior Disorders Rating Scale (DBD-RS; Pelham et al., 1992). Teacher ratings for typically developing participants were not available. Both participants with ADHD and typically developing participants completed the Wechsler Abbreviated Scale of Intelligence Second Edition (Wechsler, 2011) to obtain an estimated Full-Scale IQ.

### Final Diagnoses of ADHD and Comorbid Disorders

Best-practice methods were utilized in identifying ADHD diagnoses (Pelham et al., 2005). As such, parent and teacher rating scales Disruptive Behavior Disorder Rating Scale (DBD-RS) were utilized to identify ADHD symptoms according to DSM 5 criteria, while Impairment Rating Scale (IRS; Fabiano et al., 2006) was utilized to identify cross-situational impairment. Diagnoses of conduct disorder (CD) and oppositional defiant disorder (ODD) were determined by parental endorsement on the DBD-RS, while diagnoses of rule-out disorders were based on parent disclosure (e.g., parents reporting an autism diagnosis prior to enrollment in the study). All clinical information was reviewed by two licensed, clinical psychologists (i.e., with Ph.D.s) who determined final diagnoses of ADHD and presence of comorbid disorders (e.g., CD and ODD). A third clinician was consulted in the event that consensus could not be obtained.

### Medication Washout

All children were required to be medication free at the time of testing. Stimulant medication underwent a 24–48h washout, dependent on the type of stimulant preparation, which included 32 children with ADHD (and no typically developing participants).

### Visual Search Task Procedures

**Figure 1:**
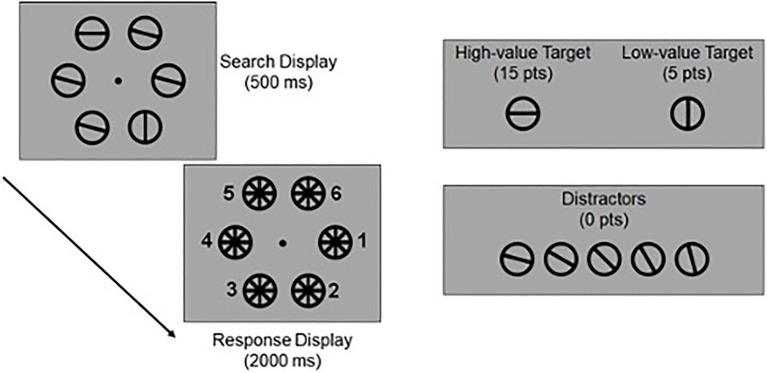
Experimental task. Stimuli are examples and not drawn to scale (Methods contain a detailed description of display parameters). See text for details.

Our experimental approach is based on a method devised by Navalpakkam et al. (2010). A trial schematic is presented in Figure 1 (note that the displays are not drawn to scale; key display parameters are provided below). Stimuli were generated in MATLAB and rendered using Psychophysics Toolbox 3.0 software extensions (Kleiner et al., 2007). Participants were seated approximately 55cm from the stimulus display (head position was not constrained). Participants made responses using the number pad on a standard US keyboard. Participants were shown a visual search display containing six oriented stimuli. Each stimulus subtended 6° visual angle (assuming a viewing distance of 55cm) with a stroke width of 0.5° and was rendered at one of six equally spaced polar locations (0–300° in 60° increments) along the perimeter of an imaginary circle (radius 6°) centered on a fixation point. Two of the four stimuli were assigned cardinal orientations (one horizontal and one vertical), while the remaining four stimuli were assigned an oblique orientation angle from the set {15, 30, 45, 60, 75}°. For each participant, we randomly selected and designated one cardinal stimulus (e.g., horizontal) as a high-value target (HVT) and the other cardinal stimulus (e.g., vertical) as a low-value target (LVT). Target designations for each participant were determined by MATLAB’s random number generator; even numbers were assigned horizontal targets as high value, while odd number was assigned vertical targets as high value. On balance assignments were fairly even across ADHD and typically developing groups: 27 of 50 ADHD subjects had vertical HVT and horizontal LVT; 27 of 48 non-ADHD had the same. These designations remained constant throughout the entire testing period. Participants were informed that they could earn points by correctly reporting the location of either the HVT or LVT on each trial *via* a keypress. Specifically, participants could earn 15 points by correctly reporting the location of the HVT on a given trial, or five points by correctly reporting the location of the LVT on a given trial. Participants received no points for reporting the location of an oblique distractor. To manipulate the relative salience of the HVT and LVT, we manipulated the orientation of the four non-target items (hereafter referred to as distractors) across trials. Specifically, on each trial, the four distractors were assigned orientations from the set {15, 30, 45, 60, 75}°. Thus, a horizontal (0°) LVT would appear conspicuous during 75° distractor trials but not during 15° trials, while the converse would be true for a vertical (90°) HVT.

The visual search display was rendered for 500ms and followed by a mask/response screen for 2,000ms. Participants were instructed to report the location of either target (i.e., HVT or LVT) by pressing the appropriate number on a computer keyboard (see Figure 1). Participants were explicitly instructed about the reward structure of the task (i.e., they could earn 15 points for correctly reporting the location of the HVT, five points for correctly reporting the location of the LVT, or zero points for reporting a distractor). Participants were instructed to prioritize accuracy over speed, but encouraged to guess if they were unsure. If the subject did not indicate a response within the 2,000ms response interval, then the trial was aborted and treated as an incorrect response. Participants were given feedback at the end of each trial (e.g., “You got the 15 point coin!”) and pressed the ENTER key to initiate the next trial when they were ready. Children were reminded that they would be awarded a small toy prize at the end of participation for positive behavior and putting forth their “best effort.” Of note, all participants earned the toy prize.

Prior to beginning the task, participants completed five practice trials to establish understanding of the task demands. Each participant completed a single block of 90 trials lasting approximately 15–20min. We quantified task performance by plotting the proportion of HVT responses as a function of feature contrast (a measure of salience defined by the ratio of the difference between the HVT, LVT, and distractors):


$$C=\frac{\left|H-D\right|}{\left|L-D\right|}$$

where *H*, *L*, and *D* are the HVT, LVT, and distractor orientations on each trial.

## Results

### Primary Analysis

#### Task Performance by ADHD Diagnostic Status

**Figure 2:**
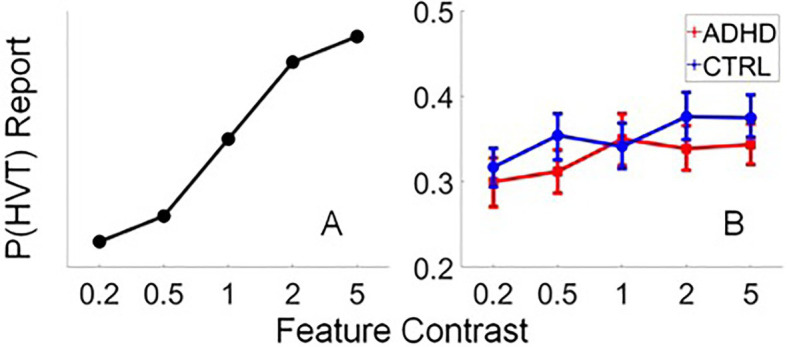
Predicted responses and pilot data. **(A)** Proportion of HVT reports by a Bayesian ideal observer plotted as a function of feature contrast, where larger values depict a greater dissimilarity between the orientation of the HVT and the distractors (see text). Data are synthetic and merely for illustrative purposes. **(B)** Proportion of HVT responses by the ADHD and control samples in the pilot dataset. Neither group resembles a Bayesian ideal observer. Error bars depict the 95% confidence interval of the mean.

An ANOVA revealed a marginal effect of diagnosis [*F*(1,96)=3.25, *p*=0.07, *η*^2^=0.033], with control participants reporting the HVT slightly more frequently than ADHD participants (*M*=0.375 vs. 0.345, respectively). There was also a marginal effect of feature contrast [*F*(4,384)=2.367, *p*=0.052, *η*^2^=0.024], but no interaction between diagnosis and feature contrast [*F*(4,384)=0.844, *p*=0.498, *η*^2^=0.009]. Figure 2A shows the predicted responses of an ideal observer during this task (note that these data are synthetic and included for illustrative purposes only). When feature contrast is low the likelihood of confusing the HVT (15 points) for a similar distractor (0 points) is high, and the optimal strategy is to report the location of the LVT (5 points) to ensure at least some reward on most trials. However, as feature contrast increases, participants should shift to reporting the HVT with more frequency to maximize rewards. The frequencies of HVT reports in the ADHD and typically developing groups tested in our pilot study are plotted as a function of feature contrast in Figure 2B. In the pilot sample, although HVT reports appear to increase with feature contrast (i.e., Figure 2B), neither group closely resembles the behavior of an ideal observer (i.e., Figure 2A).

Given the lack of support for our primary hypothesis, we examined several follow-up research questions related to group differences in other task performance metrics (e.g., reaction times, sustained attention, and performance to the task over time) and differences in performance which may be accounted for by heterogeneous factors in the ADHD group (i.e., participant age, comorbid diagnosis, and ADHD DSM 5 presentation).

### Secondary Analysis

#### Response Times

**Figure 3:**
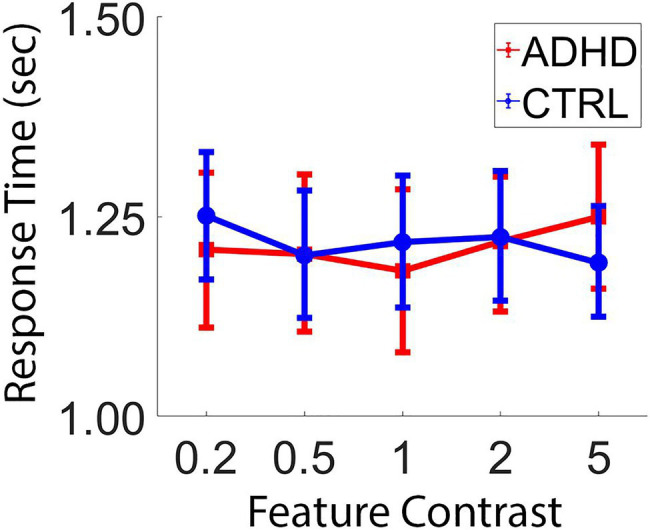
Response times. Response times for ADHD and typically developing youth according to feature contrast between targets and distractors. Error bars depict the 95% confidence interval of the mean.

Slow and variable response times (RTs) are prominent features of ADHD and have been used as an index of overall ability on tasks which require speed-accuracy tradeoffs, as prior work has shown that youth with ADHD display slower, more variable response times than typically developing youth even when accuracy is not compromised (Karalunas et al., 2012). As such, we examined group differences in response times. We observed no significant main effect of group [*F*(1,96)=0.04, *p*=0.851, *η*^2^<0.001], but did observe a significant main effect of feature contrast, [*F*(4,384)=7.13, *p*<0.001, *η*^2^=0.069], and a significant interaction of group by feature contrast [*F*(4,384)=2.99, *p*=0.019, *η*^2^=0.03]. Specifically, typically developing youth displayed slower response times when feature contrast was low and faster response times when feature contrast was high. In contrast, youth with ADHD appeared to have somewhat less consistent response times in relation to feature contrast (see Figure 3).

#### Task Duration

Prior work has also demonstrated that youth with ADHD tend to perform worse than their typically developing peers during the latter half of tasks requiring sustained attention, such as a continuous performance task (Conners et al., 1997; Rosenberg et al., 2013). As such, we examined task performance for youth with and without ADHD across time. With respect to the examination of performance on the first vs. second half of the task by feature contrast, we observed no significant main effect of time (first vs. second half) [*F*(1,49)=1.53, *p*=0.22, *η*^2^=0.303], feature contrast [*F*(4,196)=2.25, *p*=0.06, *η*^2^=0.044], nor an interaction [*F*(4,196)=2.31, *p*=0.06, *η*^2^=0.045]. We also examined performance on the first vs. second half of the task by diagnostic group and identified a non-significant three-way interaction of group, feature contrast, and time [*F*(4,384)=0.436, *p*=0.782, *η*^2^=0.005].

#### Age

We also acknowledge that task performance may be associated with development, and thus, participant age. Thus, we conducted an additional analysis examining task performance according to child age wherein we found a main effect of age [*F*(1,48)=26.43, *p*<0.0001, *η*^2^=0.36]; i.e., older children performed more optimally than younger children; however, there was no significant main effect of feature contrast [*F*(4,192)=0.743, *p*=0.564, *η*^2^=0.08] or interaction [*F*(8,380)=0.582, *p*=0.793, *η*^2^=0.012].

#### DSM 5 Presentations

Prior studies have demonstrated heterogeneous performance among youth with ADHD, even when no group differences are observed between youth with ADHD and typically developing youth (Fair et al., 2012). With respect to the examination of specific ADHD DSM 5 presentations (i.e., inattentive vs. combined), the three-way comparison between controls, ADHD-inattentive, and ADHD-combined resulted in a significant main effect of group [*F*(2,95)=3.144, *p*=0.047, *η*^2^=0.062]. Overall, the inattentive presentation was slightly more likely to choose the HVT than the either the control or combined presentation groups. However, we observed no significant main effect of feature contrast [*F*(4,380)=0.965, *p*=0.426, *η*^2^=0.010] or interaction, [*F*(8,380)=0.582, *p*=0.793, *η*^2^=0.012].

#### Comorbid ODD and CD

In terms of the contribution of comorbid ODD and/or CD diagnoses, a 3 (group – control vs. ADHD only vs. ADHD with comorbid ODD/CD)×5 (feature contrast) mixed ANOVA revealed a significant main effect of group [*F*(2,95)=3.79, *p*=0.025, *η*^2^=0.074] such that youth with ADHD and comorbid ODD/CD performed worse than either the ADHD only or control groups, but no significant ME of contrast [*F*(4,380)=0.62, *p*=0.64, *η*^2^=0.065] or interaction [*F*(8,380)=0.483, *p*=0.86, *η*^2^=0.01].

## Discussion

The findings reported by Navalpakkam et al. (2010) suggest that neurotypical human adults engage in a multitarget search (e.g., Figure 1), which is well predicted by a Bayesian ideal observer model that weights information about stimulus salience and value to maximize reward across trials. Here, we hypothesized that typically developing participants would exhibit optimality, while participants with ADHD will deviate from optimality. Although typically developing participants reported the HVT slightly more frequently than ADHD participants, overall task performance was relatively poor across both groups (i.e., the likelihood of reporting either target in each group was ~60%, where chance is 33%) with neither group resembling an optimal observer. Thus, it appears that the task was more challenging for the current sample, than the adults assessed in the original Navalpakkam et al. (2010). Of note, when examined, it was determined that participant age was associated with task performance, such that older participants performed more optimally.

Despite the lack of support for our primary hypothesis, we believe that examining competitive interactions between mechanisms computing visual salience and value – as well as deviations from optimal combinations of these factors – has the potential to reveal new insights into the neurobiology of ADHD and explain the behavioral heterogeneity observed within the disorder. As such, we examined several follow-up research questions related to group differences in other task performance metrics (e.g., reaction times, sustained attention, and performance to the task over time) and differences in performance which may be accounted for by heterogeneous factors in the ADHD group (i.e., comorbid diagnosis and DSM 5 presentation).

The examination of added task performance metrics revealed no significant effect of task duration (i.e., no differential performance for either group during the first compared to the second portion of the task), suggesting both the ADHD and typically developing groups remained engaged with the task across the roughly 15–20min duration of the task. In contrast, when examining response times, we observed a significant interaction of group by feature contrast. Specifically, typically developing youth displayed slower response times when feature contrast was small and faster response times when feature contrast was large, as expected. In contrast, youth with ADHD appeared to have somewhat less consistent response times in relation to feature contrast. This is congruent with prior work that suggests that youth with ADHD often have slower and more variable response times on tasks which require speed-accuracy tradeoffs (e.g., Karalunas et al., 2017).

With respect to heterogeneity within the ADHD group, when examining task performance according to DSM 5 presentation, we found that participants with the inattentive presentation were slightly more likely to choose the HVT than participants in either the combined presentation or typically developing group. Further, when accounting for comorbid ODD and/or CD, it was determined that participants in the ADHD with comorbid ODD/CD group performed significantly worse than youth in either the ADHD only or typically developing groups, perhaps reflecting greater severity or impairment in the comorbid group. It is important not to over interpret these effects, as subgroup sizes were relatively small. However, these initial results do support the overall promise of our general approach.

The pilot study presented here was characterized by a number of limitations that future research will need to address. Poor performance by both groups (i.e., ADHD and typically developing) when compared to adults suggests that the task may have been too difficult for participants in the 6–12 age range. Future studies will benefit from developing task stimuli that are more simplified and child-friendly. Future studies may also benefit from titrating the target-distractor orientation differences in the context of an unrewarded block in order to identify task performance ability at the individual level prior to examining the effects of rewards in order to ensure all participants are performing adequately prior to examining the effect of rewards. Importantly, ecological validity is always a concern when examining cognitive constructs in the lab and attempting to map such constructs into real-world behavior. While (to our knowledge) prior work has not systematically and empirically examined the ecological validity of the task utilized herein, this remains an important consideration for the field. Finally, ADHD is a highly heterogeneous disorder, and as such, future work will also benefit from a larger sample that will allow for a more complete examination of individual symptom domains, DSM 5 presentations, and comorbid diagnoses, as well as accounting for important factors, such as age, biological sex, comorbidity, history of medication, and more.

Limitations aside, future approaches that attempt to dissociate the contributions of salience and value to ongoing visual processing hold promise in refining and tailoring treatment targets for children with ADHD. For example, the behaviors of a classmate in close proximity may be more salient than those of the teacher at the front of the class, while the actions of the teacher may hold more long-term value in the form of a grade or behavioral reward for attending. In order to determine where to allocate attention, children need to combine both salience- and value-based information to make decisions regarding attention allocation. However, to date, it remains unknown whether children with ADHD are more influenced by salience or value when making such decisions. Cognitive neuroscience approaches focused on dissociating the contributions of salience and value to selective attention hold promise in understanding ADHD etiology and in refining and tailoring treatment targets for children with ADHD.

## Data Availability Statement

Data and analytic software described in this paper are available on the Open Sciences Framework: https://osf.io/7thsr/.

## Ethics Statement

The studies involving human participants were reviewed and approved by the Florida International University’s IRB. Written informed assent was provided by youth and written consent to participate in this study was provided by the participants’ legal guardian/next of kin.

## Author Contributions

EM and EE were involved in obtaining research funding, developing the study design, developing the study task, and formulated research questions. SM, KF, and RL contributed to recruitment, consent, collecting the data, and editing the manuscript. EE undertook data analyses and wrote the manuscript with EM and SM. EM supervised the study. All authors contributed to the article and approved the submitted version.

## Funding

This study was supported by the National Institute of Mental Health [grant numbers K23MH117280 (PI EM) and R03MH110812 (PI EM)].

## Conflict of Interest

The authors declare that the research was conducted in the absence of any commercial or financial relationships that could be construed as a potential conflict of interest.

## Publisher’s Note

All claims expressed in this article are solely those of the authors and do not necessarily represent those of their affiliated organizations, or those of the publisher, the editors and the reviewers. Any product that may be evaluated in this article, or claim that may be made by its manufacturer, is not guaranteed or endorsed by the publisher.
